# Effect of erythropoietin loading chitosan-tripolyphosphate nanoparticles on an IgA nephropathy rat model

**DOI:** 10.3892/etm.2014.1643

**Published:** 2014-03-28

**Authors:** XIAOLI ZHANG, YIN WU, KUN SUN, JING TAN

**Affiliations:** Department of Rheumatism and Nephropathy, The Third Affiliated Hospital of Xinxiang Medical University, Xinxiang, Henan 453003, P.R. China

**Keywords:** IgA nephropathy, renal function, erythropoietin, nanoparticles, chitosan-tripolyphosphate

## Abstract

The aim of the present study was to investigate the effect of erythropoietin (EPO) loading chitosan-tripolyphosphate (CS-TPP) nanoparticles on an immunoglobulin A nephropathy (IgAN) rat model. CS-TPP nanoparticles were produced from CS and TPP and EPO was loaded by mixing with the nanoparticles. The IgAN rat models were randomly divided into three groups: the CS-TPP-EPO group, CS-TPP group and EPO group. Hemoglobin (Hb), blood urea nitrogen (BUN) and creatinine (Cr) levels were measured in each group using a Biochemical Analyzer (Hitachi, Tokyo, Japan). The average size of nanoparticles was 485±12 nm and the encapsulation efficiency of EPO was 78.45%. The EPO release curve in CS-TPP-EPO nanoparticles exhibited a biphasic distribution *in vitro*. The levels of BUN and Cr in the CS-TPP-EPO group were significantly lower compared with the control group (P<0.05); however, the level of Hb in the CS-TPP-EPO group was higher compared with the other groups (P<0.05). The changes in Hb, BUN and Cr in the CS-TPP-EPO group were maintained for less than one week following the end of the treatment with CS-TPP-EPO nanoparticles. In conclusion, the CS-TPP-EPO nanoparticles had a lower toxicity compared with EPO and CS-TPP treatment. Furthermore, CS-TPP-EPO may improve the therapeutic effect in the IgAN model. This suggests that CS-TPP-EPO nanoparticles may be a potential therapeutic drug for the treatment of patients with IgAN.

## Introduction

Immunoglobulin A nephropathy (IgAN), also termed Berger’s disease, is the most common glomerulopathy worldwide and accounts for between 10 and 40% of cases of glomerulonephritis. It is a renal-limited form of glomerulonephritis, characterized by the deposition of IgA-containing immune deposits in the glomerular mesangium. In many of these conditions, IgA is deposited in the glomerulus without inducing inflammation, and this may be a clinically insignificant consequence of perturbed IgA homeostasis. The disease is particularly common in southern Europe and Asia and appears to be more common in Caucasians compared with individuals of African descent. The disease has also been reported in Native Americans, however, rarely. Patients with IgAN typically present with gross hematuria, often between 24 and 48 h following a pharyngeal or gastrointestinal infection, vaccination or strenuous exercise. Other cases are diagnosed upon detection of microscopic hematuria during routine physical examinations. Hypertension and nephrotic syndrome are unusual at presentation. Light microscopy of renal biopsy specimens typically shows mesangial expansion due to an increase in the size of the matrix and cells.

Previous studies have demonstrated that erythropoietin (EPO), which is primarily produced and released by peritubular capillary lining cells within the kidney, is able to regulate erythrocyte generation. Furthermore, EPO protects renal cells, however, the mechanism by which it does this remains to be elucidated. Although EPO is considered to be a promising candidate for the treatment of nephrological disorders, the half-life of EPO is too short to be effective for the treatment of IgAN. EPO production is stimulated by the availability of O_2_ for tissue metabolism. EPO facilitates the delivery of O_2_ by increasing the production of red blood cells. Impaired O_2_ delivery to the kidney, liver and brain may result from an increase in EPO production (1). In addition, EPO reduces apoptosis and oxidative stress in numerous pathological processes (2,3). It has been demonstrated that pretreated EPO has a marked protective effect against organ injuries, including the heart, brain and kidney (4–6). However, over-treatment with EPO results in uncontrolled proliferation of red blood cells and high blood viscosity. Attenuating the side effects of EPO treatment is an important problem in clinical studies.

Due to advances in nanotechnology, the half-life of the polypeptide drug may be increased and the side-effects attenuated. It has been previously demonstrated by Fayed *et al* (7) that poly lactic-co-glycolic acid (PLGA) nanoparticles containing EPO may significantly prolong its activity. It has also been demonstrated in the treatment of hypoxia and anemia in a newborn rat model, that the effect of treatment with EPO nanoparticles is 10 times greater compared with regular EPO treatment (8), suggesting that nanotechnology with EPO delivery is able to significantly enhance its therapeutic effects.

Chitosan (CS) is a common biodegradable multimer that exists widely in nature. It shows a high bioactivity. Previous studies have used CS containing peptides, proteins and water-soluble small molecules in numerous disease models (9–11). In the present study, CS and tripolyphosphate (TPP) nanoparticles containing EPO using an ionotropic gelation system were developed. The effect of CS-TPP-EPO nanoparticles in a rat IgAN model was then investigated.

## Materials and methods

### Preparation and characterization of the CS-TPP nanoparticles

A 1% CS solution (Mw, 550,000; deacetylation degree, 90%; Haidebei Co., Jinan, Shandong, China) with acetic acid was prepared at room temperature. Using magnetic stirring, TPP solution (China National Pharmaceutical Group Shanghai Chemical Reagent Co., Shanghai, China) was added dropwise into the CS solution using a 1 ml syringe, and the pH was adjusted to 5.7. Following 1 h, the solution was centrifuged at 30,000 × g for 10 min. The solution was washed with ethanol using a gradient concentration and then lyophilized to obtain CS/TPP nanoparticles. The CS-TPP nanoparticles were observed using a scanning electron microscope (SEM; Hitachi 2s100; Hitachi, Tokyo, Japan). The CS/TPP nanoparticles were dispersed in ethanol and the capsule size and distribution of the CS/TPP nanoparticles were measured using a particle size analyzer (Siemens, Munich, Germany).

### Determination of the encapsulation efficiency (EE) in the CS microcapsule and in vitro release assay

To develop EPO-containing nanoparticles, EPO powder (Sigma, St. Louis, MO, USA) was dissolved in CS solution (EPO and CS mass ratio of 1:1) prior to the addition of TPP. A total of 10 mg encapsulated dried CS-TPP-EPO nanoparticles were placed in 20 ml phosphate-buffered saline (pH 7.2) and agitated at 120 × g at 37 °C. A total of 0.5 ml supernatant was aspirated at 5 min intervals and the EPO levels were measured using the Coomassie blue protein assay kit. All experiments were repeated three times.

The CS/TPP nano microcapsules encapsulating rate was calculated using the following formula: EE% = (T_EPO_ - S_EPO_) / T_EPO_ × 100% (where EE is the encapsulation efficiency, T_EPO_ is the total content and S_EPO_ is the EPO content in the supernatant).

### Establishment of the rat IgAN model

A total of 30 female Sprague-Dawley rats, weighing between 160 and 200 g were purchased from the Experimental Animal Center of Xinxiang Medical University (Xinxiang, Henan, China). The present study was performed in accordance with the recommendations in the Guide for the Care and Use of Laboratory Animals from the National Institutes of Health (Bethesda, MD, USA). The animal protocol was reviewed and approved by the Institutional Animal Care and Use Committee of the Third Affiliated Hospital of Xinxiang Medical University. To generate an IgAN model, rats were administered 200 mg/kg bovine serum albumin (BSA) every other day for a total of 14 weeks. In addition, a total of 0.2 ml complete Freund’s adjuvant (including 2 mg BSA) was subcutaneously injected on day 1. A total of 0.2 ml incomplete Freund’s adjuvant (including 2 mg BSA) was intraperitoneally injected on days 14 and 28. Staphylococcal enterotoxin B (SEB; Academy of Military Medical Sciences Institute of the PLA microbial production, lot number 061030) was then intravenously injected (0.4 mg/kg) following 8–10 weeks. The alterations in histopathological staining, BUN and Cr in the IgAN model were then analyzed.

### In vivo experiments of CS-TPP-EPO

A total of 30 IgAN rats were randomly divided into three groups: the CS-TPP group treated without EPO loading, the CS-TPP-EPO group treated with packaged EPO (3,000 IU/kg) delivery nanoparticles and the EPO group treated with EPO directly. Each group was treated every other day for 2 weeks. Serum was collected every week to analyze the changes in blood urea nitrogen (BUN) and creatinine (Cr) levels using a Biochemical Analyzer (Hitachi, Tokyo, Japan).

### Statistical analysis

SPSS 16.0 (SPSS, Inc, Chicago, IL, USA) was used for statistical analysis and the data were analyzed using analysis of variance. P<0.05 was considered to indicate a statistically significant difference.

## Results

### Identification and characterization of nanoparticles

**Figure 1:**
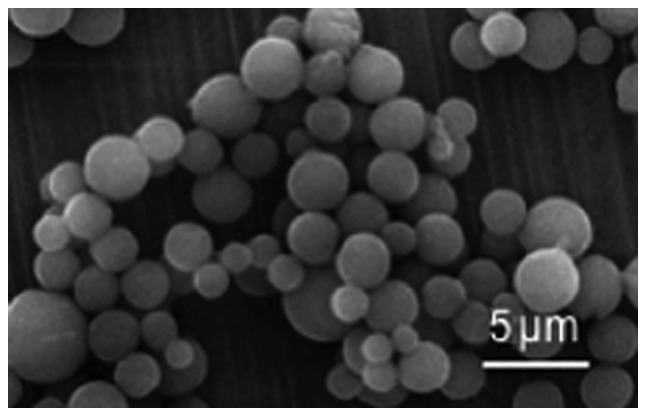
Scanning electron microscopy showing the morphology of chitosan-tripolyphosphate-erythropoietin nanoparticles.

**Figure 2:**
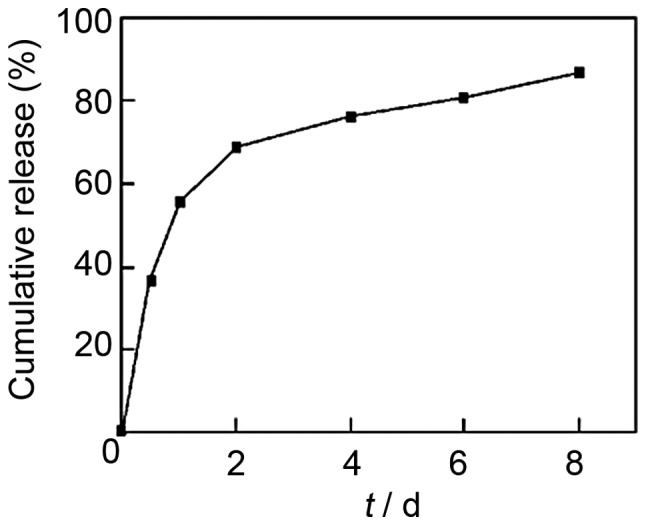
Release curve of EPO from chitosan-tripolyphosphate-EPO nanoparticles. EPO, erythropoietin.

The nanoparticles were observed using SEM. It was found that the CS-TPP-EPO nanoparticles had a smooth surface with a relatively uniform particle diameter of 485±12 nm (Fig. 1). According to the data in Table I, the amount of EPO in CS-TPP nanoparticles was raised with increasing concentrations of CS. However, the EE decreased when the concentration of CS exceeded a certain value. The release properties of CS-TPP-EPO nanoparticles with 78.45% EE were then investigated. As shown in Fig. 2, the release curve of CS-TPP-EPO microcapsules demonstrated a biphasic release: an early violent release phase and a slow release phase. Furthermore, the release of EPO from nanoparticles was sustained for up to seven days.

### Effect of CS-TPP-EPO nanoparticles in the rat IgAN model

The BUN and Cr levels in the EPO and CS-TPP-EPO groups were significantly decreased compared with the untreated rats, whilst the hemoglobin (Hb) levels were increased compared with the untreated rats. By contrast, these alterations were not observed in the CS-TPP group. The treatment of nanoparticles loaded with EPO (CS-TPP-EPO group) was more effective compared with direct EPO injection (EPO group). Although these differences were all detected between the first and third week, the changes in concentrations showed a gradient accumulation. Following the end of the treatments, the levels of BUN, Cr and Hb in the EPO group decreased slightly, whilst the levels were maintained at a stable level in the CS-TPP-EPO group (Table II).

## Discussion

Previous studies and clinical evidence have demonstrated that EPO and its derivatives are important in renal protection during chronic kidney disease. Eren *et al* (12) applied EPO treatment in rats with sepsis. The results demonstrated that EPO had a renal protective effect by reducing apoptosis (12). Another study confirmed that the synthesis of nitric oxide as a result of EPO treatment is able to protect rats with sepsis by inhibiting the nuclear factor-κB pathway (13). However, treatment with higher levels of EPO stimulates the proliferation of red blood cells, leading to an increase in blood viscosity. This results in high blood pressure and increases the risk of embolic stroke. Using an alternative mechanism of delivery of EPO, particularly via nanoparticle loading, EPO may potentially have a greater therapeutic effect (14). It has previously been demonstrated that poly lactic-co-glycolic acid (PLGA) nanoparticles containing EPO may be maintained *in vivo* for ≤14 days (7). Furthermore, EPO with PLGA nanoparticles has been used for the treatment of hypoxia and ischemia in neonatal rats (8), and improved results were found using this technology compared with traditional EPO treatment (15).

In the present study, CS and sodium TPP were used to encapsulate EPO. The size of the nanoparticles was ~485 nm and the EPO EE was 78.45%. The *in vitro* release profile of CS-TPP-EPO showed biphasic distribution, the day after the release was 50%, after a slow release, on the eighth day release was 80%. This indicates that CS-TPP nanoparticles exhibit good release properties. Since EPO is a protein with biological activity, the preparation process requires relatively mild conditions. In the present study, the condition of crosslinking reaction between CS and TPP was mild, did not react with EPO, and had the characteristic of biocompatible, sustained release resistance, low toxicity and biodegradability. Notably, in the present study the entrapment efficiency achieved a peak value at 78.45%. It was hypothesized that when the CS concentration exceeds the equilibrium value, the viscosity is also increased in parallel. Therefore, regular or spherical capsules are not formed as usual, resulting in a decrease in the EPO encapsulation rate. In addition, sustained EPO release is an important indicator for drug loading microcapsules.

In the present study, an SEB and BSA prepared rat model of IgAN was used to determine the therapeutic effect of CS-TPP-EPO on IgAN. The results demonstrated that in the CS-TPP-EPO treated group, BUN and Cr levels were significantly lower compared with the CS-TPP group, whilst the quantity of Hb increased significantly during the CS-TPP-EPO treatment. One week after treatment, BUN, Cr and blood Hb in the CS-TPP-EPO group remained at a stable concentration during treatment, however, in the EPO group the values fluctuated. This indicated that CS-TPP-EPO may sustain the release of nanoparticles *in vivo*, maintaining a high concentration of EPO in the blood and improving renal function in a rat model of IgAN.

In conclusion, to achieve an effective therapeutic effect, CS-TPP nanoparticles may be novel protein or polypeptide nanocarriers for EPO and other drugs.

## Figures and Tables

**Table I:** EE of bovine serum albumin-loading in CS/TPP nanoparticles prepared with different concentrations of CS and EPO (0.5% TPP; pH 5.7; weight ratio of CS to EPO, 1:1).

Concentration of CS (% w/v)	Weight of EPO	EE (%)
0.125	0.0375	25.10
0.25	0.75	78.45
0.5	0.15	74.63
1.0	0.3	63.22

EE, encapsulation efficiency; CS, chitosan; TPP, tripolyphosphate; EPO, erythropoietin.

**Table II:** Evaluation of BUN, Cr and Hb during the treatment at different time points.

	Week 0			Week 1			Week 2			Week 3		
				
Group	BUN (mmol/l)	Cr (μmol/l)	Hb (g/dl)	BUN (mmol/l)	Cr (μmol/l)	Hb (g/dl)	BUN (mmol/l)	Cr (μmol/l)	Hb (g/dl)	BUN (mmol/l)	Cr (μmol/l)	Hb (g/dl)
EPO	255.66±3.46	51.04±15.84	13.75±1.57	135.78±4.23	32.08±6.35	15.23±0.37	118.25±4.24	25.07±3.14	16.57±2.16	165.38±4.28	40.12±8.26	14.58±0.45
CS-TPP	238.35±4.11	49.67±12.38	13.47±1.28	252.43±4.32	53.26±5.85	12.82±3.25	258.28±3.39	55.21±6.21	11.85±0.87	264.32±3.92	57.34±10.3	11.05±0.65
CS-TPP-EPO	240.45±4.37	49.87±14.58	12.86±1.08	126.42±3.72^a^	29.92±7.04^b^	14.78±0.44^c^	120.20±3.22^a^	23.59±11.48^b^	15.25±0.58^c^	115.46±3.27^a^	22.37±12.6^b^	15.30±0.58^c^

a P<0.05,

b P<0.01,

c P<0.01 indicate a significant difference compared with week 0.

BUN, blood urea nitrogen; Cr, creatine; Hb hemoglobin; EPO, erythropoietin; CS, chitosan; TPP, tripolyphosphate.
